# Supplementary figures and images for: Response of Neuronal Populations to Phase-Locked Stimulation: Model-Based Predictions and Validation
Source: J Neurosci. 2025 Mar 11;45(15):e2269242025. doi: 10.1523/JNEUROSCI.2269-24.2025 (PMC11984083; doi:10.1523/JNEUROSCI.2269-24.2025)

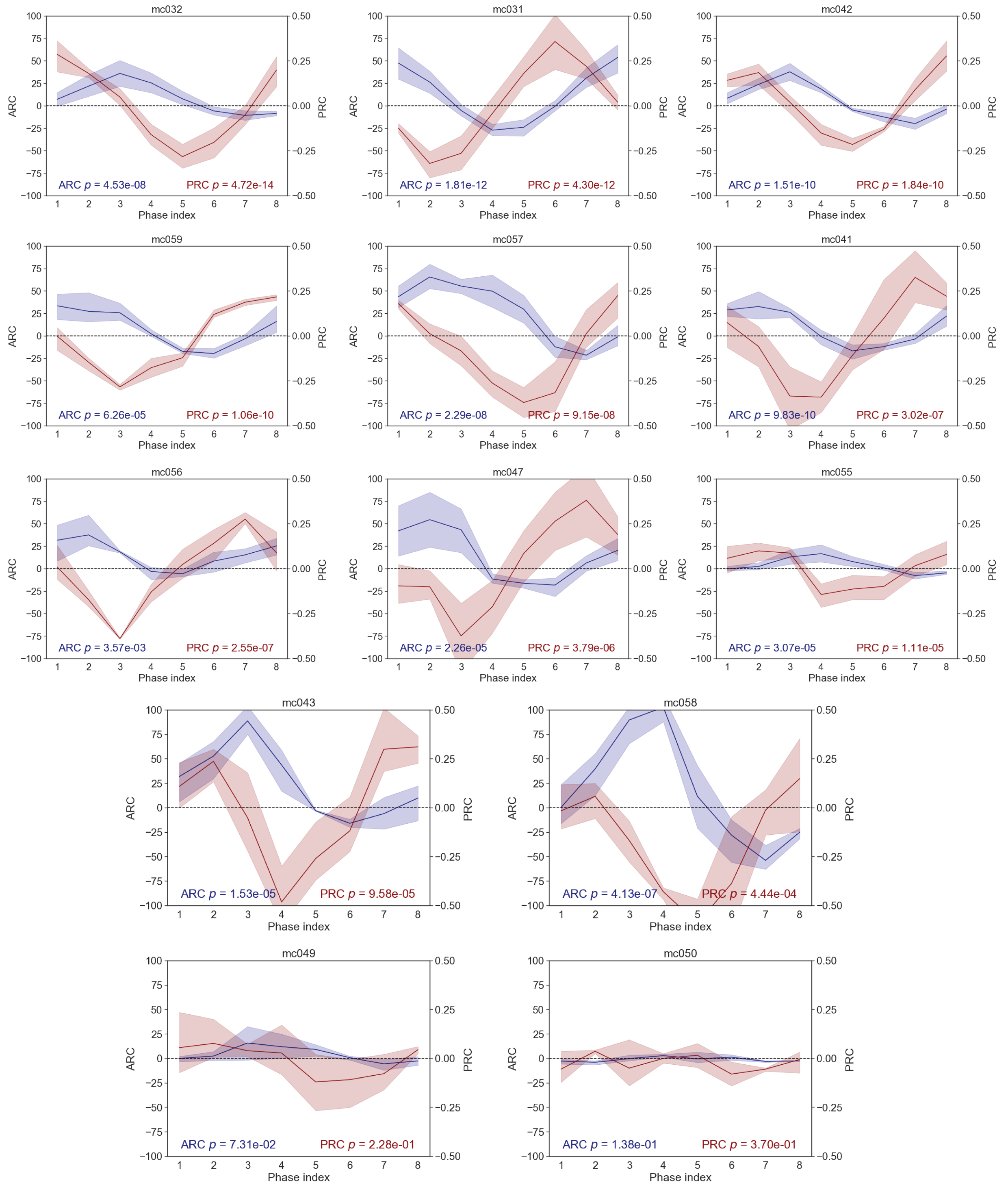

Supplement: Figure 4-1 — Response curves for individual animals (Blue: ARC, Red: PRC). For each curve, the corresponding p-values from the one-way ANOVA test are shown at the bottom. Plots are sorted according to the p-value of the PRC curves. Only two animals, mc049 and mc050, exhibit non-significant response curves. Download Figure 4-1, TIF file. [file jneuro-45-e2269242025-s001.tif]

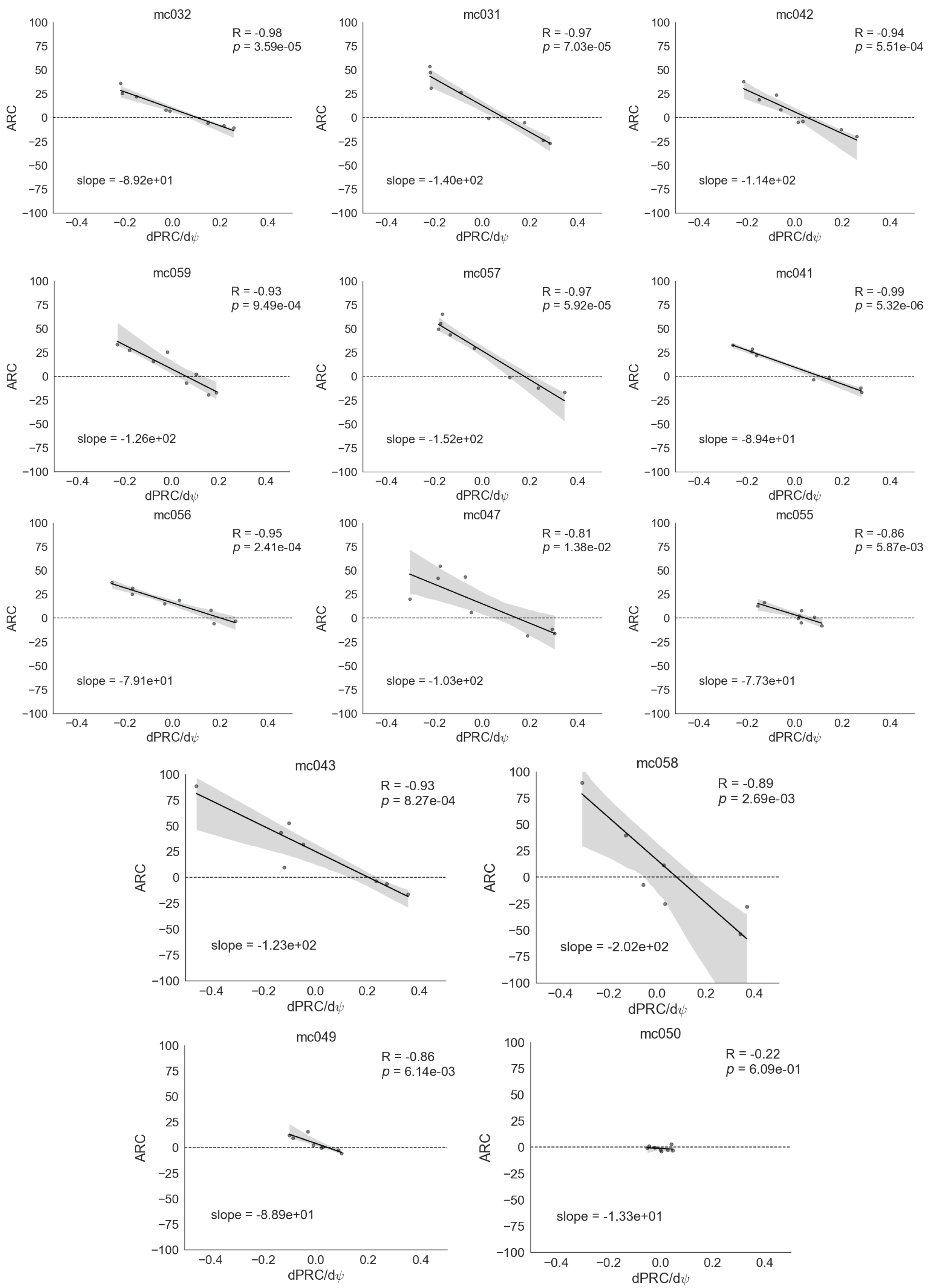

Supplement: Figure 4-2 — Correlation between the ARC and the derivative of the PRC for individual animals. Each plot displays the Pearson’s correlation coefficient, the corresponding p-value from the statistical test, and the slope of the regression line. Plots are sorted according to the p-value of the PRC curves. Download Figure 4-2, TIF file. [file jneuro-45-e2269242025-s002.tif]

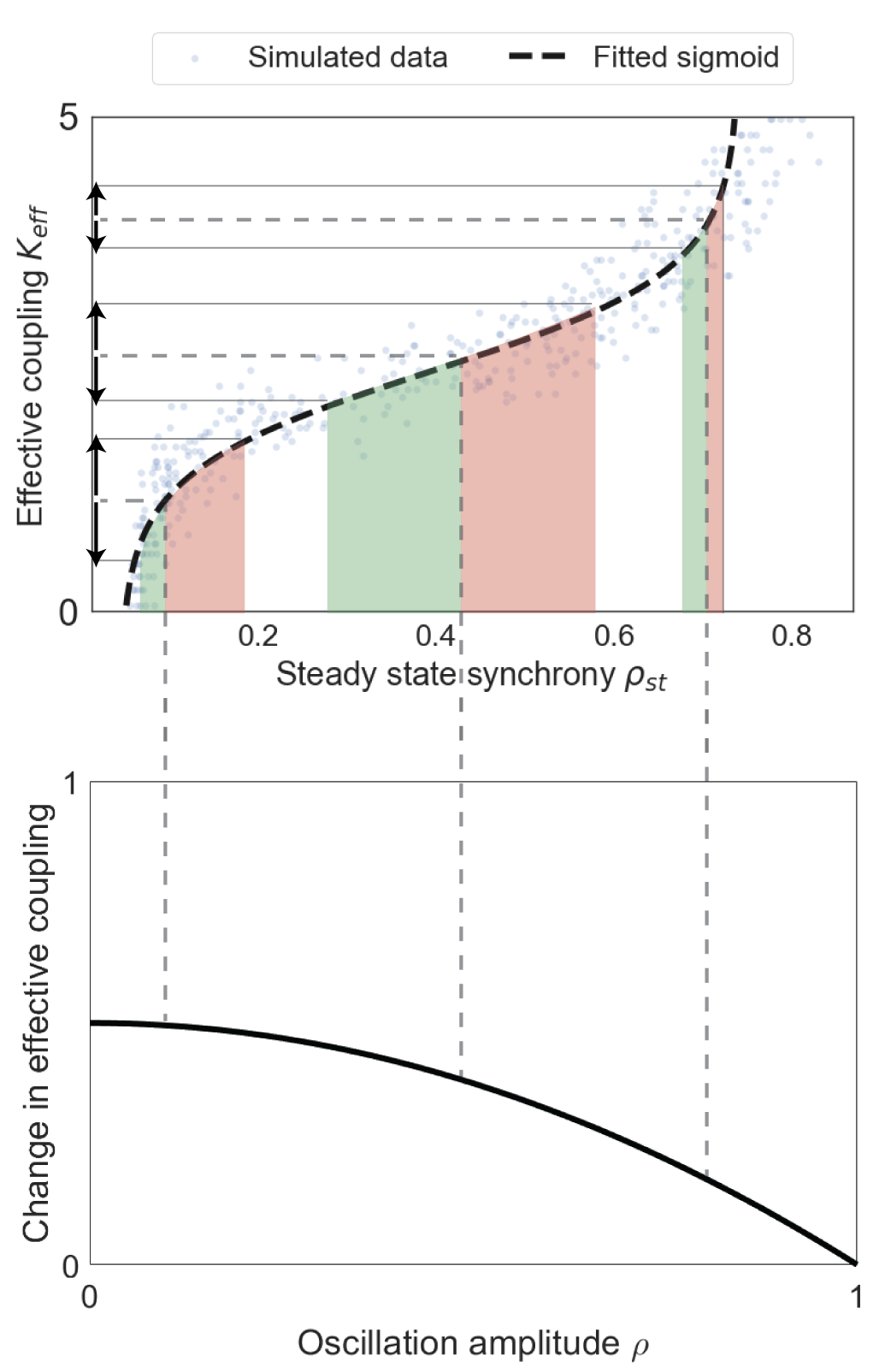

Supplement: Figure 5-1 — Role of network dynamics in the amplitude-dependence of the response illustrated by a typical operating curve of a Kuramoto network (top) and stimulation-induced disturbance as a function of oscillation amplitude (bottom). The extent of change in network synchrony as a result of stimulation depends on the size of the induced disturbance as well as network tendencies at that specific amplitude. Download Figure 5-1, TIF file. [file jneuro-45-e2269242025-s003.tif]

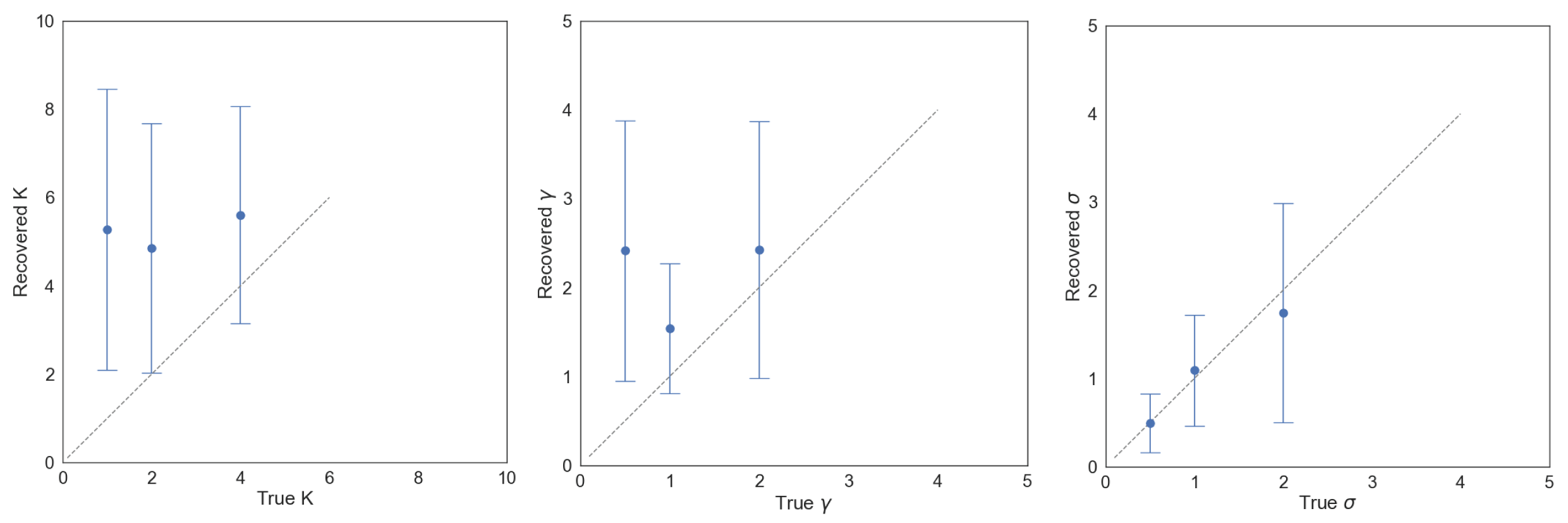

Supplement: Figure 6-1 — Parameter recovery of the Kuramoto model using synthetic data. A comparison of the true and recovered values for the intrinsic network parameters K, Γ, and σ reveals suboptimal recovery of these parameters. Download Figure 6-1, TIF file. [file jneuro-45-e2269242025-s004.tif]

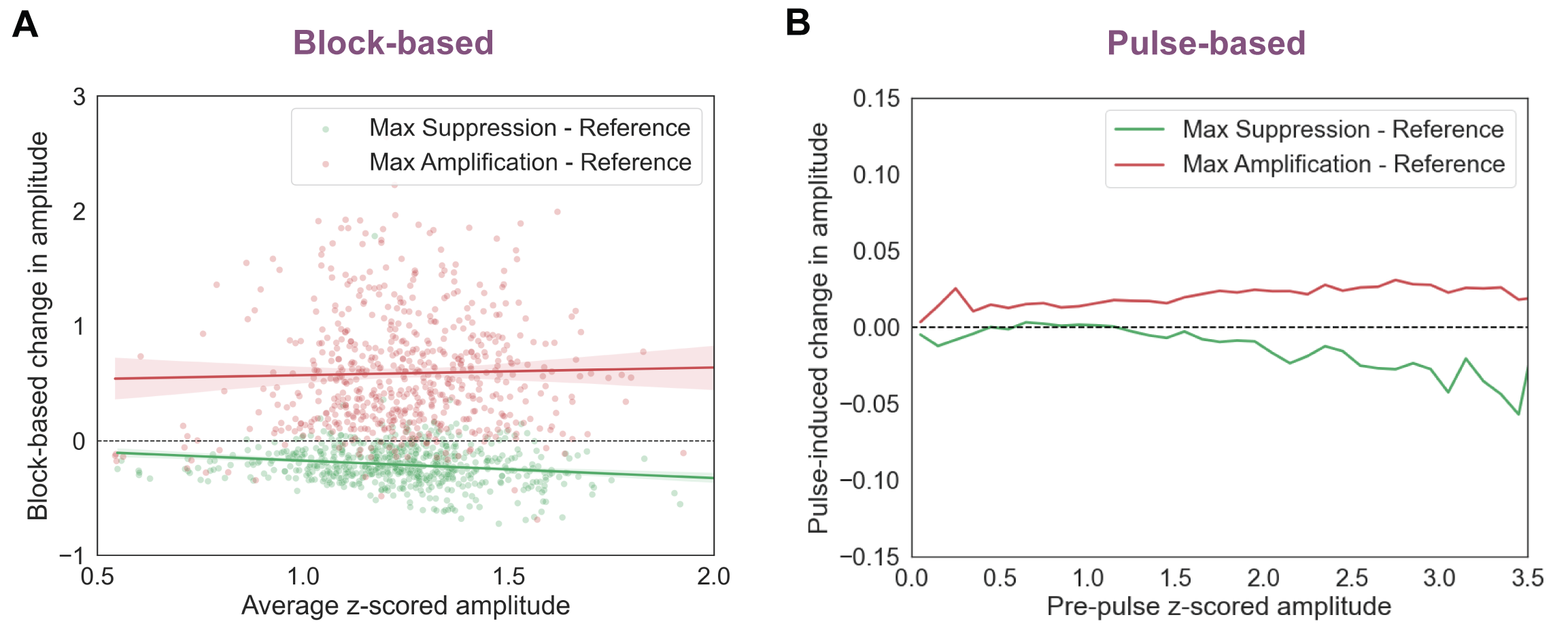

Supplement: Figure 6-3 — Reference-subtracted trends for amplification and suppression. A. Block-based approach. The regression lines for reference-subtracted values indicate significant increase of the effect (slope=-0.152, p-value=1.7e-6) in suppression versus nonsignificant change in amplification (slope=0.067, p-value=0.49). B. Pulse-based approach. Reference-subtracted curves reflect a general decay in the effect at very low synchrony values. Download Figure 6-3, TIF file. [file jneuro-45-e2269242025-s006.tif]
