# Supplementary material for: Response of Neuronal Populations to Phase-Locked Stimulation: Model-Based Predictions and Validation
Source: J Neurosci. 2025 Mar 11;45(15):e2269242025. doi: 10.1523/JNEUROSCI.2269-24.2025 (PMC11984083; doi:10.1523/JNEUROSCI.2269-24.2025)
Supplement: Figure 6-2 — Results of the model fitting for individual animals. The parameter values corresponding to the best fit at each optimization run are presented for three trials per animal. Download Figure 6-2, DOCX file. [file jneuro-45-e2269242025-s005.docx]

| **Subject** | $\boldsymbol{K}$ | $\boldsymbol{\gamma}$ | $\boldsymbol{\sigma}$ | $\boldsymbol{\omega}_{\boldsymbol{0}}$ | $\boldsymbol{f}$ | **Deviation** | **CV** |
| --- | --- | --- | --- | --- | --- | --- | --- |
| mc031 | 5.6228 | 9.1736 | 3.7082 | 224.387 | 0.0331 | 26.47515 |  |
| mc031 | 5.0726 | 9.9664 | 3.1844 | 223.9469 | 0.0357 | 25.0006 |  |
| mc031 | 5.1193 | 9.4422 | 3.5884 | 222.7461 | 0.0356 | 26.64171 | 0.028323 |
| mc032 | 5.7694 | 0.2256 | 5.786 | 225.6911 | 0.0351 | 28.1596 |  |
| mc032 | 9.4375 | 6.4934 | 4.8966 | 225.204 | 0.0345 | 27.52599 |  |
| mc032 | 4.286 | 0.6135 | 5.639 | 225.5072 | 0.0378 | 28.73932 | 0.017607 |
| mc041 | 4.8273 | 10 | 3.4075 | 223.4505 | 0.0274 | 26.78376 |  |
| mc041 | 2.6199 | 9.6109 | 3.1597 | 221.3587 | 0.0251 | 26.5856 |  |
| mc041 | 0.7454 | 9.998 | 2.6151 | 222.1537 | 0.0267 | 26.08935 | 0.011027 |
| mc042 | 4.579 | 5.0208 | 4.7925 | 224.1004 | 0.0271 | 28.43066 |  |
| mc042 | 5.1173 | 0 | 5.8324 | 222.9416 | 0.0284 | 28.89959 |  |
| mc042 | 4.5156 | 9.9736 | 3.7427 | 225.5895 | 0.029 | 29.4394 | 0.01425 |
| mc043 | 5.5455 | 9.9726 | 3.9521 | 218.0733 | 0.0341 | 30.01879 |  |
| mc043 | 6.1547 | 10 | 3.6659 | 218.4292 | 0.0336 | 27.28412 |  |
| mc043 | 2.6475 | 0.8584 | 5.5632 | 218.2556 | 0.0375 | 30.01849 | 0.044287 |
| mc047 | 6.5101 | 1.5361 | 5.7613 | 215.0154 | 0.027 | 29.75468 |  |
| mc047 | 8.0478 | 0.2487 | 5.8597 | 215.3317 | 0.0267 | 26.78568 |  |
| mc047 | 9.7847 | 10 | 4.1124 | 215.8416 | 0.0296 | 27.12713 | 0.047562 |
| mc049 | 2.5799 | 9.9541 | 3.1715 | 221.1876 | 0.0324 | 27.38671 |  |
| mc049 | 5.2138 | 9.3716 | 3.9774 | 220.747 | 0.0345 | 29.34911 |  |
| mc049 | 0.0628 | 9.0564 | 3.4165 | 219.7357 | 0.0357 | 29.72247 | 0.035549 |
| mc050 | 9.5106 | 5.4175 | 5.2621 | 218.5247 | 0.0284 | 29.0141 |  |
| mc050 | 0.7945 | 6.4858 | 3.4479 | 217.5699 | 0.0285 | 24.06511 |  |
| mc050 | 9.3705 | 4.6673 | 5.1967 | 218.3789 | 0.029 | 26.96979 | 0.0761 |
| mc055 | 0.915 | 9.6541 | 3.2064 | 249.9889 | 0.0342 | 28.6742 |  |
| mc055 | 2.4475 | 9.2304 | 3.6434 | 250 | 0.0325 | 29.28766 |  |
| mc055 | 0.0054 | 6.7924 | 3.9697 | 249.0729 | 0.0349 | 29.33792 | 0.010369 |
| mc056 | 7.8124 | 4.3539 | 5.3867 | 248.5024 | 0.0329 | 29.91194 |  |
| mc056 | 8.3298 | 10 | 4.1165 | 250 | 0.0325 | 28.61577 |  |
| mc056 | 2.4395 | 8.6916 | 4.0708 | 250 | 0.0349 | 31.51511 | 0.03951 |
| mc057 | 1.3925 | 9.9441 | 1.4411 | 217.2204 | 0.0393 | 20.57247 |  |
| mc057 | 6.2647 | 1.148 | 5.3233 | 219.1022 | 0.0431 | 24.36882 |  |
| mc057 | 1.3622 | 9.9063 | 1.9662 | 219.1962 | 0.0409 | 22.31634 | 0.069207 |
| mc058 | 2.3443 | 6.0666 | 3.564 | 217.7543 | 0.0431 | 22.491 |  |
| mc058 | 0 | 8.0559 | 2.4053 | 216.5805 | 0.0435 | 21.89727 |  |
| mc058 | 0.0006 | 9.9595 | 0.7317 | 217.5308 | 0.0439 | 20.45372 | 0.039581 |
| mc059 | 8.511 | 10 | 4.4737 | 250 | 0.0453 | 31.50299 |  |
| mc059 | 1.1251 | 5.7165 | 4.1351 | 249.9951 | 0.0454 | 27.40695 |  |
| mc059 | 7.502 | 2.6516 | 5.4681 | 250 | 0.0433 | 27.70132 | 0.064612 |
